# Effect of low-level laser therapy on the post-surgical inflammatory process after third molar removal: study protocol for a double-blind randomized controlled trial

**DOI:** 10.1186/1745-6215-14-373

**Published:** 2013-11-06

**Authors:** Simone Oliveira Sierra, Alessandro Melo Deana, Raquel Agnelli Mesquita Ferrari, Priscilla Maia Albarello, Sandra Kalil Bussadori, Kristianne Porta Santos Fernandes

**Affiliations:** 1 Postgraduate Program in Rehabilitation Sciences, Universidade Nove de Julho (UNINOVE), Rua Vergueiro, 235, São Paulo, SP CEP: 01504-001, Brazil; 2 Postgraduate Program in Biophotonics Applied to Health Sciences, Universidade Nove de Julho (UNINOVE), Rua Vergueiro, 235, São Paulo, SP CEP: 01504-001, Brazil

**Keywords:** Laser, Inflammation, Repair, Tooth extraction, Randomized controlled trial

## Abstract

**Background:**

Low-level laser therapy (LLLT) has been shown to modulate the inflammatory process without adverse effects , by reducing pain and swelling and promoting the repair of damaged tissues. Because pain, swelling and muscle spasm are complications found in virtually all patients following oral surgery for the removal of impacted teeth, this model has been widely used to evaluate the effects of LLLT on the inflammatory process involving bone and, connective tissue and the muscles involved in mastication.

**Methods/Design:**

After meeting the eligibility criteria, 60 patients treated at a Specialty Dental Center for the removal of impacted lower third molars will be randomly divided into five groups according to the type of laser therapy used at the end of surgery (intraoral irradiation with 660 nm laser; extraoral irradiation with 660 nm laser; intraoral irradiation with 808 nm laser; extraoral irradiation with 808 nm laser and no irradiation). To ensure that patients are blinded to the type of treatment they are receiving, the hand piece of the laser apparatus will be applied both intraorally and extraorally to all participants, but the device will be turned on only at the appropriate time, as determined by the randomization process. At 2 and 7 days after surgery, the patients will be evaluated by three blinded evaluators who will measure of swelling, mouth opening (muscle spasm evaluation) and pain (using two different pain scales). The 14-item Oral Health Impact Profile (OHIP-14) will be used to assess QOL. All data will be analyzed with respect to the normality of distribution using the Shapiro-Wilk test. Statistically significant differences between the experimental groups will be determined using analysis of variance, followed by a suitable *post hoc* test, when necessary. The significance level will be set at α = 0.05.

**Discussion:**

The lack of standardization in studies with regard to the samples, methods and LLLT parameters complicates the determination of the actual effect of laser therapy on this model. The present study aims to provide a randomized, controlled, double-blind trial to compare four different LLLT parameters in relation to the outcomes of pain, swelling and muscle spasm following surgery for the extraction of impacted third molars and evaluate the effects os surgery on patients' quality os life (QOL).

**Trial registration:**

Brazilian Registry of Clinical Trials - Rebec (RBR-6XSB5H).

## Background

Low-level laser therapy (LLLT) has been shown to modulate the inflammatory process without adverse effects, by reducing pain and swelling and promoting the repair of damaged tissues [1,2]. The effect of LLLT on acute pain from a soft-tissue injury may be related to the consequent reduction in edema, hemorrhage, neutrophil infiltration, inflammatory cytokines and enzymes [3]. The swelling-reduction effect of LLLT may be related to its ability to accelerate the regeneration of lymph vessels and decrease vascular permeability [4-6].

A large number of reports exist regarding the effect of LLLT on the tissue repair process, especially the inflammatory processes that affect muscle tissue [7-10]. However, studies addressing the effects of LLLT on muscle spasms caused by the inflammatory process have reported conflicting results [11-17].

Because the removal of impacted third molars involves damage to bone, and connective tissue and the muscles involved in mastication, this model has been widely used to evaluate the effect of LLLT on the inflammatory process [1,18,19]. Indeed, a considerable number of studies have evaluated the effect of LLLT on reductions in pain, swelling and muscle spasm following the surgical removal of impacted third molars, but the lack of standardization in the methods and dosimetric parameters used has compromised evaluation of the desired outcomes and hinders the acceptance of LLLT as an effective method for minimizing the adverse effects of third molar surgery [1].

In the literature, eight articles have assessed pain [11,12,15-17,20-22]. Only studies that used intraoral application of red laser irradiation reported a reduction in postoperative pain, but the parameters were not fully described in any of these articles [20,21].

With regard to swelling [11-17,22,23], a reduction in postoperative edema was obtained in one study that used red laser (50 mW, 4 J/cm^2^) applied intraorally [23], one that used infrared laser (100 mW, 12 J, 4 J/cm^2^) extraorally [14] and two that used infrared laser (100 mW, 12 J, 4 J/cm^2^ and 300 mW, 54 J, respectively) with a combination of intraoral and extraoral irradiation [13,17].

Concerning muscle spasm [11-17], a reduction was found in one study that used red laser (300 mW, 10 J/cm^2^) intraorally [16], two studies that used infrared laser (100 mW, 120 12 J, 4 J/cm^2^ and 300 mW, 54 J) both intraorally and extraorally [13,17], and one study that used infrared laser (100 mW, 12 J, 4 J/cm^2^) either extraorally or intraorally [14].

The aim of the proposed project is to carry out a randomized, controlled, double-blind, clinical trial evaluating the effects of LLLT on pain, swelling and muscle spasm following surgical removal of impacted third molars. Comparisons will be made of two sites (intraoral versus extraoral) and different laser wavelengths (red versus infrared).

## Methods/Design

### Study location

This randomized, controlled, double-blind, clinical trial will be carried out in the Specialty Dental Center of the city of São Bernardo do Campo, state of São Paulo, Brazil.

### Study design and composition of study sample

The study design will consist of five treatments evaluated on two occasions (at 2 and 7 days following surgery). Table 1 shows the scheme for repeated-measures analysis of variance. Because the measures of pain, swelling and mouth opening have low variability when using the proposed scales, 40 degrees of freedom will be sufficient to control for residual variance. Thus, a minimum sample size of 50 subjects (10 in each group) is sufficient. Thus 50 patients undergoing treatment at the center for the removal of impacted lower third molars will participate in the study.

**Table 1:** Scheme for repeated-measures analysis of variance

**Causes of variation**	**Degrees of freedom**
Treatment	4
Period	1
Treatment × period	4
Residual	40
Total	49

**Figure 1:**
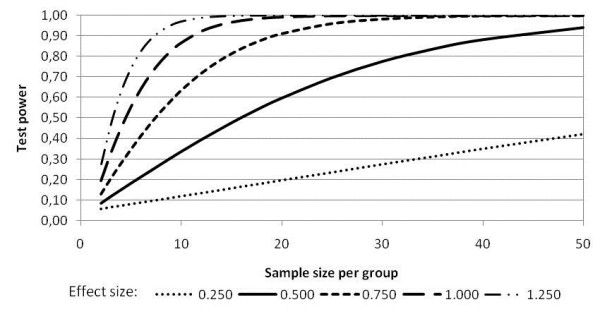
The power analysis.

In addition, as this work predicts a two-factor (two wavelength), two-group (two irradiation sites) analysis, there will be 20 patients in each group (10 in each experimental subgroup) and 20 in control group. The power analysis (Figure 1) shows that for medium and large effect size, the test power will remain above 0.8 for 20 subjects in each group.

### Ethics approval

The study has received approval from the UNINOVE Human Research Ethics Committee (protocol number 15410 and 34248) and is registered with both the World Health Organization (Universal Trial Number U1111-1129-9338) and the Brazilian Registry of Clinical Trials (RBR-6XSB5H).

### Inclusion criteria

Patients undergoing surgical removal of impacted lower third molars will be included in the study if they agree to participate after reading and signing a statement of informed consent.

### Exclusion criteria

The exclusion criteria include: presence of systemic disease, chronic pain or neurological/psychiatric disorder; current smoking habit; use of anti-inflammatory agent, analgesic or bisphosphonate drug in the previous 15 days; pericoronitis in the previous month; pregnancy or current breastfeeding; or history of photosensitivity disorders.

### Randomization and composition of groups

**Figure 2:**
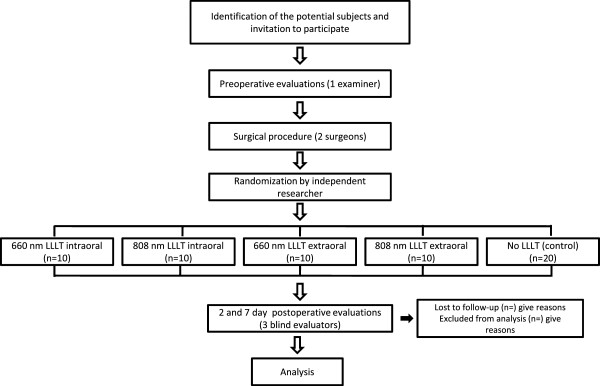
Flowchart characterizing the experimental design, sample composition, and trial protocol.

After undergoing a clinical evaluation by the dental surgeons, patients who meet the eligibility criteria will be divided into five experimental groups (Figure 2) based on a randomization method involving raffle numbers. Randomization will be conducted by a researcher not involved in the recruitment and treatment of the participants. Concealed allocation will be performed using a set of random numbers placed in sealed opaque envelopes. The laser operator will open the envelope containing the procedure to be performed on each patient immediately following third molar surgery. Sealed envelopes awaiting new subjects will be kept in a safe place and given to the operator as the sessions are scheduled. The 40 patients will be allocated into four experimental groups or a control group as follows: Group 1 (660 nm laser, applied intraorally, n = 10), Group 2 (808 nm laser, applied intraorally, n = 10), Group 3 (660 nm laser, applied extraorally, n = 10), Group 4 (808 nm laser, applied extraorally, n = 10), Group 5 (control; no irradiation, n = 20).

### Blinding procedures

The surgical procedures will be performed by two dental surgeons and a third person will perform both the preoperative evaluation and LLLT. Following a calibration exercise, three blinded examiners, who have not previously been involved in the evaluation, surgery or laser therapy, will perform the postoperative evaluations. The patients will be unaware of the group to which they are allocated.

### Experimental protocol

#### Preoperative evaluations

##### Personal data

Information on gender (male/female), ethnicity (Caucasian, mixed race or African descent), education (illiterate to completed postgraduate) and age (years) will be collected prior to surgery by the laser operator.

##### Surgical difficulty

The difficulty of the surgical procedure will be determined based on the Winter Classification, the Pell and Gregory classification and the modified Prant Classification. The Winter Classification [24] considers the alignment of the impacted tooth (vertical, horizontal, mesioangular, disto-angular, horizontal vestibular, or inverted). The Pell and Gregory scales [25] consider the position of the tooth on the occlusal plane (on a scale of A to C) and the ascending ramus of the mandible (on a scale of 1 to 3). The Prant scale modified by Amarillas-Escobar *et al.*[15] classifies the surgical procedure on a five point scale (grade I - extraction with forceps only, grade II - extraction by osteotomy, grade III - extraction by osteotomy and coronal section, and grade IV - complex extraction. At the end of the surgical procedures, the surgeons will classify the procedures and will record the duration of each operation from incision to final suture.

##### Facial measurements

Prior to surgery, the laser operator will measure and record for each patient the distances between the corner of the eye and angle of the mandible, between the tragus and the lip commissure, and between the tragus and pogonion as described by Amarillas-Escobar *et al.*[15].

##### Mouth opening

Prior to surgery, mouth opening will be assessed by the laser operator, using a caliper to measure the distance between the incisal edges of the upper and lower central incisors as described previously [11,13,14,17].

### LLLT

#### Instrument

The Photon Laser III GaAlAs (DMC, São Carlos, São Paulo, Brazil) will be used. The active medium are a Arsenide-Gallium-Aluminium and a Indium-Gallium- Aluminium- Phosphide semiconductor diodes. Emission is in the red and near -infrared wavelengths, with variable power values in the continuous emission mode. The display provides the dosage according to the power and application time.

#### Irradiation parameters

The laser operator will perform the LLLT for each patient immediately following third molar surgery using the parameters given in Table 2.

**Table 2:** LLLT parameters

**Parameter**	**Red laser**	**Infrared laser**
Center wavelength [nm]	652	808
Spectral bandwidth (FWHM) [nm]	5	2.6
Operating mode	Continuous wave	Continuous wave
Average radiant Power [mW]	100	100
Polarization	Random	Random
Aperture diameter [cm]	0.094	0.094
Irradiance at aperture [mW/cm^2^]	3537	3537
Beam profile	Multimode	Multimode
Beam spot size at target [cm^2^]	0.02827	0.02827
Irradiance at target [mW/cm^2^]	3537	3537
Exposure duration [s]	30	30
Radiant exposure [J/cm^2^]	106	106
Radiant energy [J]	3	3
Number of points irradiated	4	4
Area irradiated [cm^2^]	0.113	0.113
Application technique	Contact	Contact
Number and frequency of treatment sessions	1 session	1 session
Total radiant energy [J]	12	12

Intraoral irradiation will be performed by positioning the laser probe directly in contact with four points on the gingival mucosa in the area of the surgical field: point 1, – middle of the bone socket; 2, - the cervical third of the lingual face; 3, - the middle third of the lingual face; and 4 - apical third of the lingual face. The laser will be applied for 30 seconds for each point.

Extraoral irradiation will be performed by positioning the laser probe in contact with the skin on four points of the masseter muscle: 1 –, lower region (near the mandibular insertion); 2 –, lower middle region; 3 –, upper middle region; and 4 –, upper region (near the insertion of the zygomatic arch). Again, the laser will be applied for 30 seconds for each point.

#### Postoperative evaluations

##### Evaluation of postoperative pain

Because postoperative pain following third molar extraction reaches its maximum intensity within 3 to 5 hours, continues for 2 to 3 days and gradually decreases until the postoperative day 7, this outcome will be assessed 2 and 7 days following surgery [18] using a visual analog scale (VAS) and the Numeric Rating Scale 101 (NRS-101). The VAS is a 10 cm linear scale, ranging from 0 (no pain) to 10 (worst possible pain), while the NRS-101, measures pain on a scale ranging from 0 (no pain) and 100 (worst possible pain). The patients will be instructed by one of the post-surgical evaluators to mark a point on the VAS, indicating the intensity of the pain [12,15,22] and for the NRS-101, to attribute a number between 0 and 100 that best represents the pain they are experiencing [11,20].

##### Evaluation of postoperative swelling

Postoperative swelling reaches a peak 12 to 48 h following third molar extraction and begins to decrease during the subsequent days, disappearing around 5 to 7 days postoperatively [19,23,26,27]. To measure swelling, most authors measure the distance between two [11,13,14,23] or three predetermined anatomical points on the face [15]. In the present study, the same three evaluators mentioned above will measure the distances between the corner of the eye and angle of the mandible, between the tragus and lip commissure and between the tragus and pogonion of each patient 2 and 7 days following surgery.

##### Evaluation of postoperative muscle spasm

Spasms in the muscles of mastication can limit or even prevent mouth opening following surgical removal of impacted third molars [11-17]. This outcome is usually assessed by measuring the distance between the incisal edges of the upper and lower central incisors using a caliper [11,13,14,17]. In the present study, the same three evaluators will measure mouth opening in each patient at 2 and 7 days following surgery.

##### Evaluation of presence and intensity of hematoma/ecchymosis

The presence of hematoma/ecchymosis will be evaluated by measuring the largest diameter of any color changes in the skin of the cheek and submandibular region at 2 and 7 days after surgery. The measure will be performed by the same three evaluators, who will classify the occurrence of this outcome into four categories: 1) no color changes; 2) spot diameter of less than 4 cm; 3) spot diameter between 4 and 10 cm; and 4) spot diameter greater than 10 cm, as described by Bjornsson *et al*. [28].

##### OHIP-14 questionnaire

The 14-item Oral Health Impact Profile (OHIP-14) is a simplified form of the original OHIP questionnaire used for evaluating of the effect of oral health status on quality of life (QOL). The items are classified into the following subscales: functional limitation, pain, psychological discomfort, physical disability, psychological disability, social disability and handicap. The questionnaire will be administered to the patients by the same three evaluators 7 days following surgery.

##### Patients’ feelings concerning their postoperative status

The same three evaluators will ask the patients the following 10 questions at 2 and 7 days after surgery:

1) Are you maintaining your normal social activities?

2) Are you working/studying normally?

3) Are you maintaining your normal diet?

4) Have you had difficulty swallowing because of the surgery?

5) Have you had difficulty tasting foods?

6) Can you chew on the operated side?

7) Have you had trouble sleeping because of the surgery?

8) Have you had difficulty speaking because of the surgery?

9) Has your appearance changed because of the surgery?

10) Have you experienced nausea since the surgery?

### Evaluation of results

The patients will be evaluated 2 and 7 days after surgery with regard to the three primary outcomes: pain (VAS and NRS-101), swelling (comparison of preoperative and postoperative facial measurements) and muscle spasms (comparison of preoperative and postoperative mouth opening). The following data will also be recorded, as these are frequently analyzed in postoperative evaluations [1,15,24,25,29-33]: degree of surgical difficulty (Pell and Gregory classification, Winter classification and modified Prant classification); number of cartridges used for anesthesia; occurrence of hemorrhage during surgery; duration of surgery (minutes) from initial incision to final suture; and presence of hematoma/ecchymosis (largest diameter of color changes in the skin of the cheek and submandibular region). Individual variables (gender, ethnicity, educational level and age), OHIP-14 score and effect of surgery on QOL using the 10-item questionnaire will be evaluated, as suggested by other authors [29,31-36].

### Statistical analysis

All data will be analyzed with respect to the normality of distribution using the Shapiro-Wilk test. Statistically significant differences between the experimental groups will be determined using analysis of variance, followed by a suitable post hoc test, if necessary. The significance level will be set at α = 0.05. By assuming normality in the distribution of the data, two-way ANOVA offers a high power for the design of this trial.

## Discussion

Because virtually all patients experience pain, swelling, and muscle spasm as complications found in virtually all patients following oral surgery for the removal of bone and teeth (especially third molars) and these symptoms have a profound effect on QOL in the first few days after surgery [29-36] this model has been widely used to evaluate the effect of LLLT on the inflammatory process involving bone, connective tissue and the muscles involved in mastication [1,11-17,20-23]. In addition, removal of impacted third molars is one of the most common procedures in oral surgery [1,34,36]. However, the lack of standardization in studies with regard to the samples, methods and LLLT parameters complicates the determination of the actual effect of laser therapy on this model [1]. The aim of the present study is to use a randomized, controlled, double-blind trial to compare four different LLLT parameters in relation to the outcomes of pain, swelling and muscle spasm following surgery for the extraction of impacted third molars.

## Trial status

At the time of submission of the manuscript, the study is in the data collection phase.

## Competing interests

The authors declare no conflicts of interests.

## Authors’ contributions

KPSF, SOS and ADM provided the idea for the study, established the hypothesis and wrote the original proposal. KPSF, RAMF and PMA made significant contributions to drafting the paper. SKB and ADM performed critical revision of the manuscript. All authors reviewed and approved the final manuscript.
